# Cerebral Energy Status and Altered Metabolism in Early Brain Injury After Aneurysmal Subarachnoid Hemorrhage: A Prospective 31P-MRS Pilot Study

**DOI:** 10.3389/fneur.2022.831537

**Published:** 2022-02-28

**Authors:** Stephanie Alice Treichl, Wing Mann Ho, Ruth Steiger, Astrid Ellen Grams, Andreas Rietzler, Markus Luger, Elke Ruth Gizewski, Claudius Thomé, Ondra Petr

**Affiliations:** ^1^Department of Neurosurgery, Medical University of Innsbruck, Innsbruck, Austria; ^2^Department of Neuroradiology, Medical University of Innsbruck, Innsbruck, Austria; ^3^Department of Anesthesiology and Critical Care Medicine, Medical University of Innsbruck, Innsbruck, Austria; ^4^Neuroimaging Research Core Facility, Medical University of Innsbruck, Innsbruck, Austria

**Keywords:** brain metabolism, early brain injury, subarachnoid hemorrhage, 31-P-MR-spectroscopy, energy status

## Abstract

**Background:**

Acute changes of cerebral energy metabolism in early brain injury (EBI) after aneurysmal subarachnoid hemorrhage (aSAH) may play a crucial role for overall neurological outcome. However, direct detection of these alterations is limited. Phosphorous magnetic resonance spectroscopy (31P-MRS) is a molecular-based advanced neuroimaging technique allowing measurements of pathophysiological processes and tissue metabolism based on various phosphorous compound metabolites. This method may provide objective assessment of both primary and secondary changes.

**Objective:**

The aim of this pilot study was to evaluate the feasibility and the diagnostic potential of early 31P-MRS in aSAH.

**Methods:**

Patients with aSAH treated for ruptured aneurysms between July 2016 and October 2017 were prospectively included in the study. 3-Tesla-MRI including 31P-MRS was performed within the first 72 h after hemorrhage. Data of the vascular territories of the anterior, middle, and posterior cerebral arteries (ACA, MCA, PCA) and the basal ganglia were separately analyzed and compared with data of a healthy age- and sex-matched control group. Phosphorous compound metabolites were quantified, and ratios of these metabolites were further evaluated. Influence of treatment modality, clinical conditions, and analgosedation were analyzed.

**Results:**

Data of 13 patients were analyzed. 31P-MRS showed significant changes in cerebral energy metabolism after aSAH in all cerebrovascular territories. Both PCr/ATP and PCr/Pi ratio were notably increased (*P* < 0.001). Also, Pi/ATP was significantly decreased in all cerebrovascular territories (*P* = 0.014). PME/PDE ratio was overall significant decreased (*P* < 0.001).

**Conclusion:**

31P-MRS is a promising non-invasive imaging tool for the assessment of changes in energy metabolism after aSAH. It allows a detailed insight into EBI and seems to harbor a high potential for clinical practice.

## Introduction

Aneurysmal subarachnoid hemorrhage (aSAH) and the associated early brain injury (EBI) are accompanied by a complex series of secondary pathophysiological cascades, leading to global alterations of the cellular environment and energy metabolism (1–3).

Detecting these changes in cerebral energy metabolism and mitochondrial dysfunction might be crucial for further treatment and assessment of overall neurological outcome, yet to date it is inconceivable only with the use of conventional neuroimaging methods. Multimodal cerebral neuromonitoring (i.e., brain tissue oxygenation and cerebral microdialysis) represents one of the sporadic options to identify clinically relevant pathophysiological alterations and their development. However, these methods estimate changes only locally, with no possibility to evaluate the complex situation in the entire brain (4–6). Moreover, multimodal cerebral monitoring is an invasive neurosurgical procedure associated with potentially relevant surgery-related complications (7).

In contrast, magnetic resonance spectroscopy (MRS) allows non-invasive measurements of selected metabolites, in case of 31P-MRS phosphorous compound metabolites (8), potentially providing comprehensive insights into posthemorrhagic pathophysiological processes with objective assessment of tissue damage, altered cerebral metabolism, and “tissue at risk” in a non-invasive manner. Furthermore, the early phase after aSAH and the underlying cerebral energy metabolic changes have not yet been sufficiently clarified.

To the best of our knowledge, there are only few clinical series employing 31P-MRS in aSAH, all with small numbers of patients and variable time points when 31P-MRS was performed. Interestingly, in animal models of aneurysm rupture, numerous changes in energy metabolism have been reported, however with inconsistent findings (9–12) and limited transferability to humans.

The aim of our study was to evaluate energy metabolism in EBI (<72 h) after aSAH using 31P-MRS to detect relevant energy alterations, additionally compared to age- and sex-matched healthy controls.

We therefore prospectively analyzed 13 consecutive aSAH patients with ruptured intracranial aneurysms who underwent an early MRI including 31P-MRS performed within the first 72 h after SAH in order to thoroughly evaluate all cerebral areas and cerebrovascular territories, to detect changes of cerebral energy metabolism, gaining a better understanding of complex pathophysiological processes during EBI after aSAH.

## Materials and Methods

Patients older than 18 years of age with aSAH who were admitted to our Department between July 2016 and October 2017, were prospectively included in the study. Depending on clinical parameters and aneurysm morphology, patients underwent microsurgical clipping or endovascular coil embolization after interdisciplinary decision and the intervention was carried out within 24 h. An early MRI was performed within 72 h after the hemorrhagic event. Exclusion criteria were contraindications to MRI (e.g., pacemaker, metal objects), or an unstable clinical condition disqualifying the patient to undergo an MRI examination safely. As the effect of mechanical ventilation and analgosedation on cerebral metabolism assessed by MRS has not yet been thoroughly clarified, patients were dichotomized according to sedation and ventilation for analysis.

The effect of treatment modality remains unclear, as a craniotomy is considered more invasive and may possibly have an additional influence on cerebral metabolism. Besides, given the anatomical and location differences between the surgically and endovascularly treated aneurysms, we additionally performed a statistical subanalysis with patients dichotomized by the treatment modality.

A total of 13 patients met the inclusion criteria and were prospectively enrolled in the study. Detailed demographic data are summarized in Table 1. Upon admission, aSAH patients were evaluated with computed tomography (CT) and CT angiography (CTA), complemented with cerebral catheter angiography in most cases. Also, invasive cranial multimodal neuromonitoring was inserted if indicated and further ultra-early aneurysm treatment routinely undertaken within the first hours after admission. As mentioned above, all MRI scans were performed within 72 h after SAH. Of note, the mean arterial blood pressure was continuously kept at a level of >80 mmHg, as it is standard in the management of aSAH patients at our site to ensure adequate cerebral perfusion. To achieve this, norepinephrine was administered continuously through a central venous catheter and the dose was adjusted accordingly. Midazolam and sufentanil were administered for standardized general analgosedation.

**Table 1:** Demographic data of all included patients.

13 patients	Male	2	
	Female	11	
Age (years)	54.5	Range	34–70
H&H class	1	7	
	2	2	
	3	3	
	4	1	
	5	0	
Treatment	Surgical	9	
	Endovascular	4	
Localization	ACOM	5	
	ACI	1	
	MCA	2	
	PCOM	3	
	DACA (pericallosal artery)	2	
EVD/monitoring	None	6	
	EVD/monitoring	7	

*ACOM, anterior communicating artery; ACI, internal carotid artery; MCA, middle cerebral artery; PCOM, posterior communicating artery; DACA, distal anterior cerebral artery or pericallosal artery; EVD, external ventricular drain*.

To allow comparison of cerebral energy metabolism with a healthy population, 13 healthy control subjects were matched (age/gender) and analyzed. All MRI examinations were performed using the same 3 Tesla device (see below). The study was approved by the Local Ethics Committee of the Medical University Innsbruck (Protocol number: AN2016-0032 359/4.8). Written informed consent was obtained according to Austrian law either from the patient when regaining consciousness and legal capacity, or from her/his guardian.

### Magnetic Resonance Imaging

**Figure 1:**
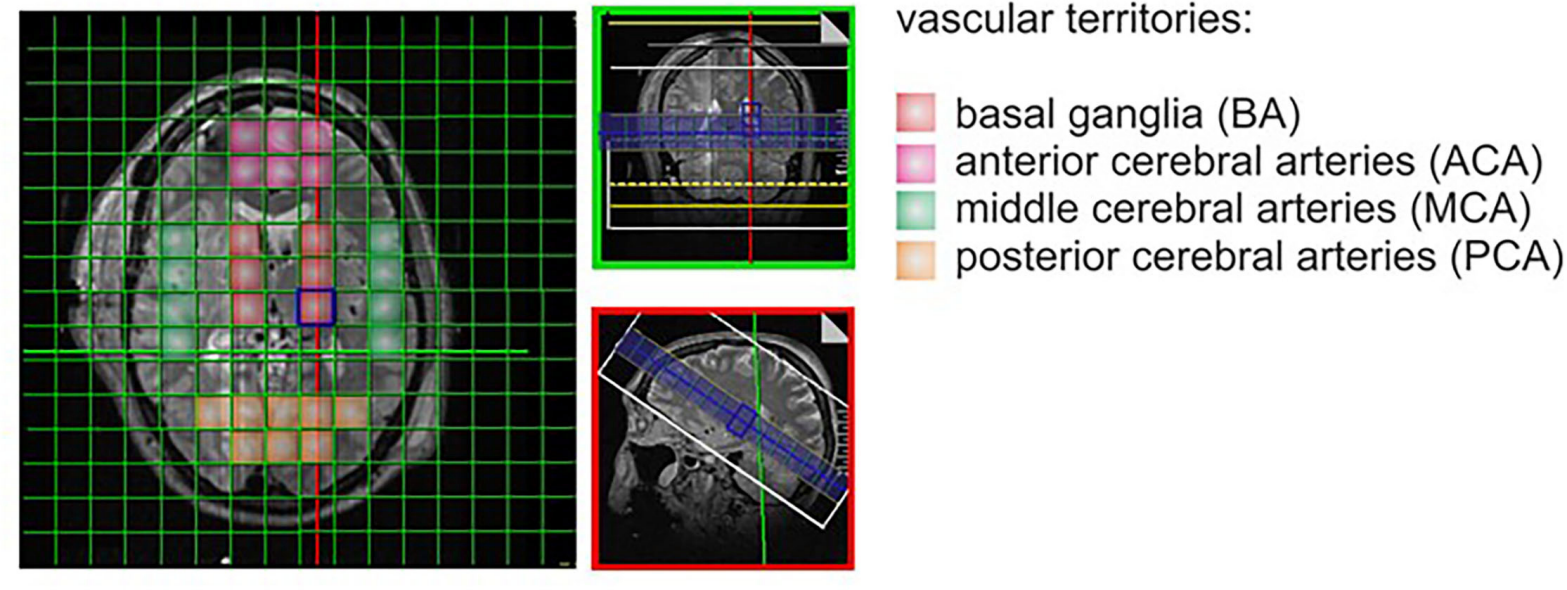
Spectroscopy with voxel.

All patients underwent standard MRI performed on a 3T whole-body system (Verio, Siemens Medical AG, Erlangen, Germany). 31P-MRS was performed on the same MRI unit with a double-tuned 1H/31P volume head coil (Rapid Biomedical, Würzburg, Germany). For each patient, one MRS 3D block based on a previously acquired T2 space sequence of the entire brain was recorded, resulting in an extrapolated 16 × 16 × 8 matrix and a field of view of 240 × 240 mm^2^, with a single voxel size of 15 × 15 × 25 mm^3^. For 31P-MRS data evaluation, voxels of interest were selected from all cerebrovascular territories (ACA, MCA, PCA, and basal ganglia separately). See Figure 1.

In the healthy control group, data designated for comparison were collected from the same cerebrovascular territories and selected areas of matched patients anatomically corresponding with the voxels of interest. 31P-MRS data were processed offline with the jMRUI software package (version 5.0, available at: http://www.mrui.uab.es), utilizing the non-linear least square fitting algorithm AMARES (13). Phospho-creatine (PCr), adenosine-tri-phosphate (ATP), free phosphate (Pi), phosphomonoesther (PME), and phosphodiesther (PDE) were determined from each voxel of interest. Although still a matter of debate, defined metabolite ratios have been introduced as surrogate parameters for crucial processes in cellular metabolism (14). Accordingly, ratios representing ATP resynthesis (PCr/ATP), ATP hydrolysis (Pi/ATP), energy demand (PCr/Pi), and membrane turnover (PME/PDE) were used and calculated for the structurally affected tissue as well as the aforementioned cerebrovascular territories and these ratios were compared with the corresponding brain regions of the healthy control group (15–17).

Importantly, absolute values of phosphorus metabolites may not be simply used for reliable interpatient comparison given the significant differences between the individuals. Thus, various ratios of phosphorous metabolites are routinely used here. Accordingly, we interpreted PCr/ATP-ratio as an indirect measure of ATP resynthesis as previously described by Wallimann et al. (17), Pi/ATP ratio as ATP hydrolysis, PCr/Pi ratio as a tissue energy demand and PME/PDE ratio as a membrane turnover as described by Liu et al. (14).

### Statistical Analysis

Statistical analysis was conducted using IBM SPSS Statistics (V.21, version 21, SPSS Inc., IBM, Chicago, IL, USA). Continuous variables were reported as mean values and confidence intervals. The calculated ratios were tested for normality using the Shapiro-Wilk test. Also, ANOVA was performed to check for significant differences. Differences with a *P*-value of <0.05 were considered statistically significant.

## Results

A total of 13 patients (aged 34–70 years, mean 54.5 years; 11 female and 2 male patients) with aSAH were enrolled in the study. The most common initial clinical presentation according to the Hunt&Hess classification and WFNS classification was grade 1 in seven patients (53.8%), followed by grade 2 in two patients (15.4%), and grade 3 in three patients (23.1%). One patient presented with Hunt&Hess grade 4 (7.7%). Detailed demographic data are summarized in Table 1.

**Figure 2:**
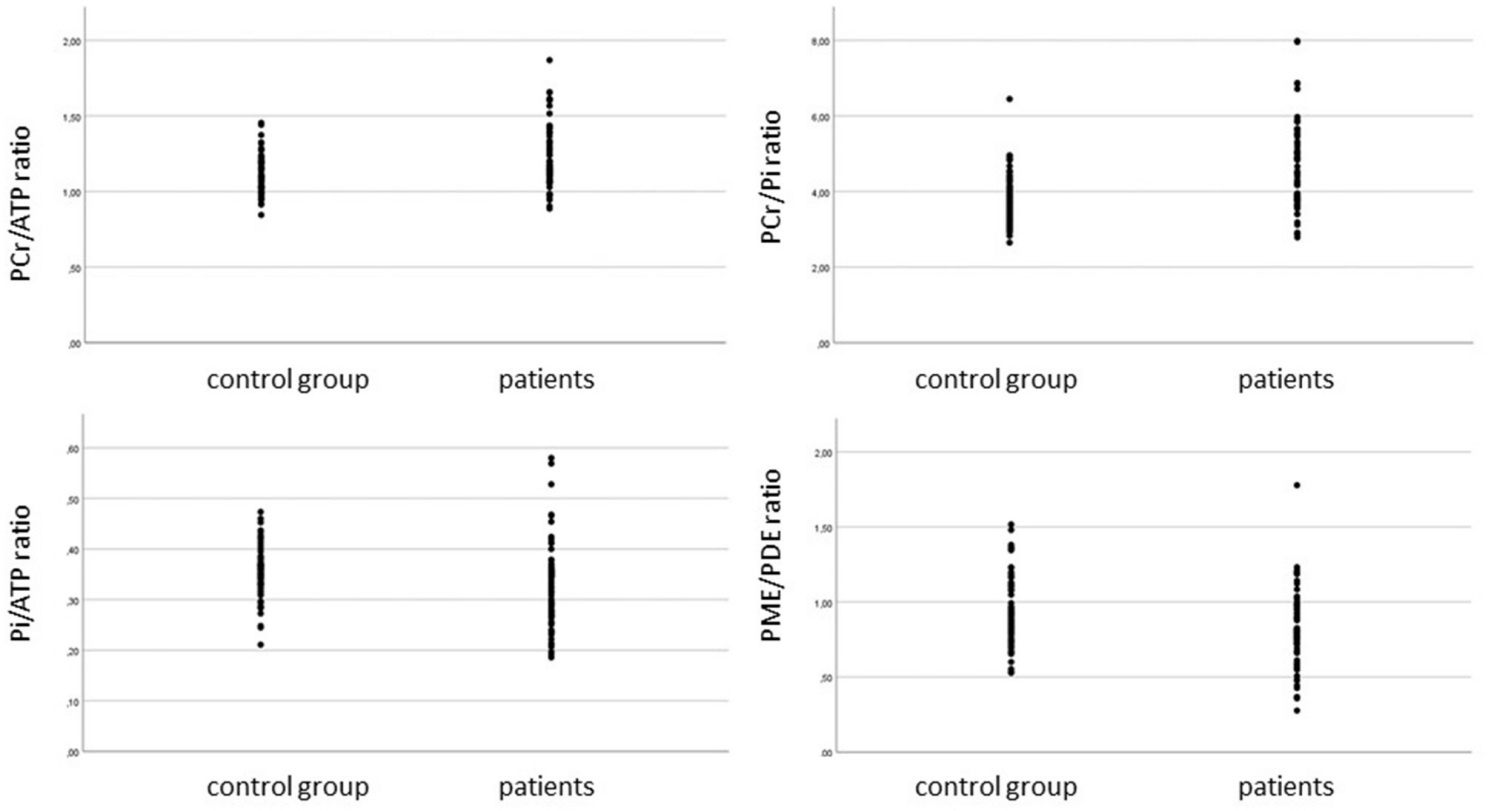
PCr/ATP ratio, PCR/Pi ratio, Pi/ATP ratio, and PME/PDE ratio in SAH patients and control group. ANOVA performed.

After aneurysmal SAH, there was a globally increased PCr/ATP ratio in all ROIs representing the state of ATP resynthesis (*P* < 0.001). Pi/ATP ratio reflecting the ATP hydrolysis conditions was notably decreased (*P* = 0.014), resulting in global impairment of ATP utilization in all cerebrovascular territories. PCr/Pi ratio representing tissue energy demand was significantly increased after aSAH in all cerebrovascular territories (*P* < 0.001). PME/PDE ratio standing for membrane turnover significantly different between the healthy control group and aSAH patients, favoring the healthy controls (*P* < 0.001). Results are summarized in Table 2 and seen in Figure 2.

**Table 2:** ANOVA, ratios of phosphorous metabolites.

**Overall**	**Patients mean (CI)**	**Controls mean (CI)**	***P*-value**
PCr/ATP	1.1238 (1.1780–1.2698)	1.1098 (1.0824–1.1371)	*P* < 0.001
PCr/Pi	4.5417 (4.2975–4.7859)	3.7722 (3.6305–3.9140)	*P* < 0.001
Pi/ATP	0.3273 (0.3096–0.3450)	0.3531 (0.3424–0.3638)	*P* = 0.014
PME/PDE	0.8099 (0.7557–0.8640)	0.9377 (0.8867–0.9886)	*P* < 0.001

*PCr, Phosphocreatine; Pi, inorganic phosphate; ATP, adenosine triphosphate; PME, phosphomonoester; PDE, phosphodiester*.

Given the potential impact of analgosedation and mechanical ventilation on 31-P-MRS results, we dichotomized our patients into two groups. Three patients (23.1%) were analgosedated and mechanically ventilated at time of MRI examination, 10 patients (76.9%) underwent the MRI awake. There were significant alterations in the following ratios between the groups performing ANOVA: PCR/ATP ratio (*p* < 0.001), PCR/Pi ratio (*p* < 0.001), Pi/ATP ratio (*P* = 0.049), and PME/PDE ratio (*P* < 0.001).

**Figure 3:**
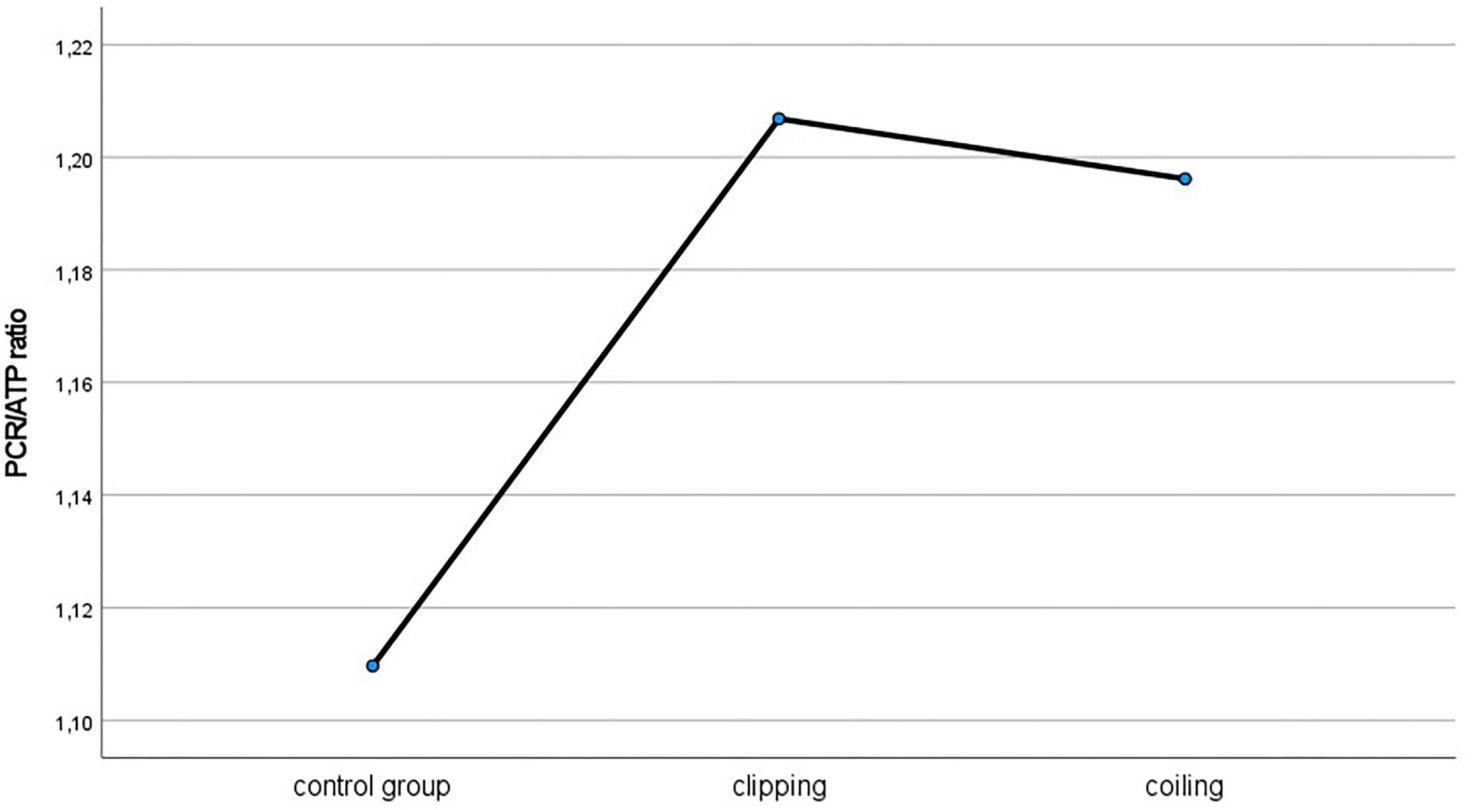
PCr/ATP ratio in patients treated microsurgically and endovascularly compared to healthy control group.

When considering the used treatment modality for the ruptured aneurysms, there were apparent changes in all PCR/ATP, PCR/Pi, Pi/ATP, and PME/PDE ratios between the healthy control group and all treated patients (see the Figure 3). Yet, energy cerebral metabolism did not differ between the surgically and endovascularly treated patients.

**Figure 4:**
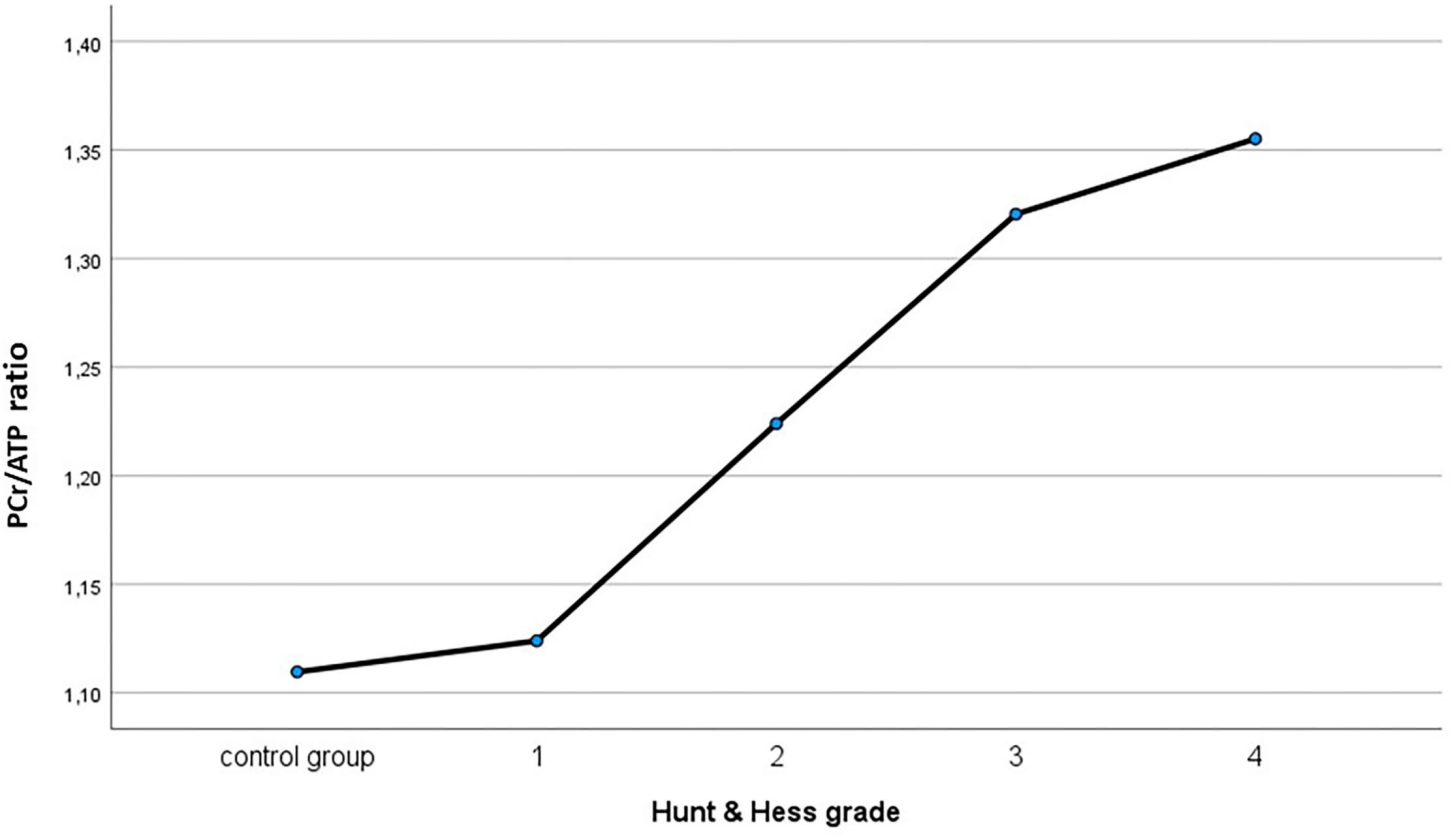
PCr/ATP ratio analyzed according to initial clinical presentation compared to healthy control group.

Importantly, all changes in the aforementioned ratios proportionated to both severity of aSAH and initial clinical patient condition. Performing ANOVA, there were impairments in PCr/ATP ratio (*P* < 0.001), PCr/Pi ratio (*P* < 0.001), Pi/ATP ratio (*P* = 0.011), and PME/PDE ratio (*P* = 0.017). Results are summarized in Figure 4 and Table 3.

**Table 3:** ANOVA, analysis regarding initial clinical presentation, treatment modality, analgosedation, and ventilation and inserted EVD and monitoring.

	**PCR/ATP ratio**	**PCR/Pi ratio**	**Pi/ATP ratio**	**PME/PDE ratio**
Cerebrovascular territories	*P* < 0.001	*P* < 0.001	*P* = 0.011	*P* = 0.017
Treatment modality	n.s	n.s	n.s	n.s
Analgosedation and mechanical ventilation	*P* < 0.001	*P* < 0.001	*P* = 0.049	*P* < 0.001
EVD and monitoring	n.s	n.s	n.s	n.s
Initial clinical presentation	*P* < 0.001	*P* < 0.001	*P* = 0.031	*P* < 0.001

*PCr, Phosphocreatine; Pi, inorganic phosphate; ATP, adenosine triphosphate; PME, phosphomonoester; PDE, phosphodiester; EVD, external ventricular drain*.

## Discussion

This prospective pilot study of 13 patients with aSAH undergoing early MRI within 72 h after hemorrhage provides first comprehensive data on 31P-MRS in EBI after aSAH. These indicate highly significant global alterations in cerebral energy metabolism in all cerebrovascular territories independently of aneurysm location.

Compared to healthy controls there were several significant alterations in cerebral energy metabolism in the acute stage of aSAH. We found a substantial increase of the PCr/ATP ratio representing a lacking resynthesis of ATP in aSAH patients alongside with an altered membrane turnover, expressed as a lower PME/PDE ratio in contrast to the healthy control group. Interestingly, our data indicate an impaired ATP degradation after aSAH, reflected by substantially decreased Pi/ATP ratio differences between the SAH-affected patients and the healthy age- and sex-matched control group. Energy demand was accordingly reduced, represented by an increased PCr/Pi ratio. This implies major disturbances of the cerebral energy metabolism, not only in terms of ATP synthesis but also at the level of ATP utilization in the entire brain. These findings of decreased Pi/ATP ratios in EBI due to aSAH appear to be similar to them after traumatic brain injury (TBI) as shown in human subjects by Garnett et al. (18). Likewise, Pinggera et al. found recently similar changes in brain metabolism in the acute state in after severe TBI (19, 20). Stovell et al. analyzed PCr/ATP ratios in a small group of patients in the acute state after traumatic TBI using 31P-MRS and correlated these findings with tissue NADH/NAD+ after succinate supplementation (21). They found no global significant changes in PCr/ATP ratios yet a subgroup of patients with mitochondrial dysfunction presented with increased PCr/ATP ratio In contrast, in our current study, PCr/ATP ratio was substantially increased in all cerebrovascular territories, strongly indicating a complex cerebral energy metabolism impairment including disturbed ATP resynthesis in acute brain injury after aneurysmal SAH. Likewise, numerous cerebral metabolic functions seem to be impaired after TBI also in animals as shown by Yoshino et al. In their rat model simulating cerebral concussion with no evidence of direct morphological damage the authors studied several metabolic processes within the first 10 days after TBI. They reported an early rather global metabolic depression which lasted for as long as 5 days. Their results indicated dramatic alterations in cell metabolic functioning (22).

31P-MRS obviously allows insight into cerebral energy metabolism in a non-invasive way. In defiance of the challenging character of MRI in critically ill patients and possible technical difficulties associated with required MR-compatibility, we have demonstrated that early MRS after aSAH is technically feasible, representing a promising non-invasive tool for comprehensive unbiased assessment of brain damage and posthemorrhagic “tissue at risk.”

In our study, mean arterial blood pressure was continuously kept above 80 mmHg in order to eschew hypotension-induced hypoperfusion and its resultant potential distortion of the findings. There were no relevant MRI-related adverse events or clinical deterioration. Likewise, the safety and feasibility of advanced MRI examinations in critically ill patients have been recently affirmed in 26 patients with severe TBI, who underwent an early MRI without any additional risk or morbidity (23).

A decrease of PCR/ATP levels has been described in a rat model with induced SAH-like events (11). In a monkey model Handa et al. showed a reduction of ATP during induced hypotension and therefore suspected a correlation between changes in brain metabolism and a reduced CBF due to impaired autoregulation (12). Schubert et al. found a significant reduction of CBF within the first 12 h after aSAH examined with Xenon-CT in humans, which was independent from CPP and ICP (24). This hypoperfusion correlated with the initial clinical presentation, i.e., SAH grade, but was present globally even in low-grade SAH. Interestingly, our 31P-MRS measurements also demonstrated a global affection of the brain, even though the majority of patients presented with lower SAH grades being in good clinical condition. Early brain injury appears thus characterized by global cerebral metabolic changes. We found the cerebral metabolism altered not only in voxels adjacent to the ruptured aneurysm but also in all other cerebrovascular territories.

Our results indicate an increased ratio of PCr/Pi, so that aSAH most likely escalates global energy demand in the acute phase of the disease. Steiger et al. showed a decreased Pi/PCr ratio in less perfused areas in a patient with giant cell arteritis and high-grade stenosis of the vertebral artery and the internal carotid artery (25). Based on our apparent findings we esteem 31P-MRS for a promising non-invasive tool how to assess pertinent metabolic changes in all cerebrovascular territories at a subcellular level, and not only locally depending on neuromonitoring probe positioning. Importantly, the interpretation of our findings must be performed carefully due to the small number of the included patients. Yet, we assume that the differences may become even more apparent in case of larger study group.

31P-MRS is being routinely used to examine metabolic changes in other intracranial lesions such as tumors or psychiatric diseases (26–28). Also, some animal studies investigated TBI and subsequent effects on brain metabolism as well as on magnesium levels (29, 30). Ishige et al. showed an overall decrease of ATP and PCr after TBI in a rat model (31). Similarly, several clinical series examined trauma-related changes in energy metabolism and magnesium levels in humans (32–34). For example, Garnett et al. performed 31P-MRS in TBI patients after a mean of 9 days (range 2–21 days) and found a significant decrease of pH and magnesium in cerebral tissue as well as elevated ratios of PCr/Pi and PCr/ATP, while the Pi/ATP ratio was decreased (18).

It is worthy of notice that MRI in ventilated patients in an early stage is highly challenging and related to numerous critical care issues (23). Furthermore, to date it has not been sufficiently elucidated yet whether and how mechanical ventilation and analgosedation commonly applied in both preclinical and clinical studies affect brain oxygenation and cerebral energy metabolism and the related 31-P-MRS findings under the analgosedation. In our study we found substantial global changes in the cerebral energy metabolism in both intubated and conscious patients indicating that the 31P-MRS is feasible and reliable also under the terms of anesthesia.

We acknowledge that our study has several limitations. Certainly, the results must be interpreted carefully in the light of the small sample size evaluated. A comparison of alterations between the hemispheres or an investigation of the possible impact of aneurysm location were thus only finite. Additionally, the voxel size is rather large, making a differentiation between gray and white matter arduous. Also, it is worth noticing that the majority of the included patients in our pilot study presented with low-grade hemorrhage. This represents a certain bias in patient selection for high-grade SAH patients present with rather unstable clinical conditions and the advanced MRI examinations remain challenging in the neurointensive care patients (19).

It may have resulted in possible distortion of our findings toward difference mitigation between the SAH patients and the healthy control group. Given the current apparent differences in our study, if including also the high-grade hemorrhages one may expect even stronger divergence between the groups.

The limitations notwithstanding, our findings and the results from the aforementioned previous studies produced consistent results and comparable data.

## Conclusion

The highly significant changes in cerebral energy metabolism in all cerebrovascular territories mirrors the complexity of EBI after aneurysmal SAH, indicating a global impairment of cerebral metabolism at multiple levels. This prospective clinical trial using 31P-MRS identifies MRS as a possible promising advanced neuroimaging technique in the acute stage of SAH. It may provide a better understanding and a more detailed insight into the pathophysiological metabolic processes and interactions after aneurysmal SAH in a non-invasive fashion. Yet, future studies are needed with larger number of patients.

## Data Availability Statement

The raw data supporting the conclusions of this article will be made available by the authors, without undue reservation.

## Ethics Statement

The studies involving human participants were reviewed and approved by Local Ethics Committee of the Medical University Innsbruck (Protocol Number: AN2016-0032 359/4.8). The patients/participants provided their written informed consent to participate in this study.

## Author Contributions

ST, AG, CT, and OP: conception or design of the work. ST, WH, RS, AR, and OP: data collection. ST, WH, RS, AG, AR, EG, CT, and OP: data analysis and interpretation. ST and OP: drafting the article. All authors critical revision of the article and final approval of the version to be published.

## Conflict of Interest

The authors declare that the research was conducted in the absence of any commercial or financial relationships that could be construed as a potential conflict of interest.

## Publisher's Note

All claims expressed in this article are solely those of the authors and do not necessarily represent those of their affiliated organizations, or those of the publisher, the editors and the reviewers. Any product that may be evaluated in this article, or claim that may be made by its manufacturer, is not guaranteed or endorsed by the publisher.

## References

[1] van Lieshout JH Dibué-Adjei M Cornelius JF Slotty PJ Schneider T Restin T. An introduction to the pathophysiology of aneurysmal subarachnoid hemorrhage. Neurosurg Rev. (2018) 41:917–30. 10.1007/s10143-017-0827-y 28215029

[2] Fujii M Yan J Rolland WB Soejima Y Caner B Zhang JH. Early brain injury, an evolving frontier in subarachnoid hemorrhage research. Transl Stroke Res. (2013) 4:432–46. 10.1007/s12975-013-0257-2 23894255 PMC3719879

[3] Ostrowski RP Colohan AR Zhang JH. Molecular mechanisms of early brain injury after subarachnoid hemorrhage. Neurol Res. (2013) 28:399–414. 10.1179/016164106x115008 16759443

[4] Stocchetti N Roux PL Vespa P Oddo M Citerio G Andrews PJ. Clinical review: neuromonitoring - an update. Crit Care. (2013) 17:201–201. 10.1186/cc11513 23320763 PMC4057243

[5] Oddo M Villa F Citerio G. Brain multimodality monitoring. Curr Opin Crit Care. (2012) 18:111–8. 10.1097/mcc.0b013e32835132a5 22322259

[6] de Lima Oliveira M Kairalla AC Fonoff ET Martinez RC Teixeira MJ Bor-Seng-Shu E. Cerebral microdialysis in traumatic brain injury and subarachnoid hemorrhage: state of the art. Neurocrit Care. (2014) 21:152–62. 10.1007/s12028-013-9884-4 24072457

[7] Tavakoli S Peitz G Ares W Hafeez S Grandhi R. Complications of invasive intracranial pressure monitoring devices in neurocritical care. Neurosurg Focus. (2017) 43:E6. 10.3171/2017.8.focus17450 29088962

[8] Kemp GJ. Non-invasive methods for studying brain energy metabolism: what they show and what it means. Dev Neurosci Basel. (2000) 22:418–28. 10.1159/000017471 11111158

[9] Altura BM Gebrewold A Altura BT Gupta RK. Role of brain [Mg2+]i in alcohol-induced hemorrhagic stroke in a rat model: a 31P-NMR *in vivo* study. Alcohol. (1995) 12:131–6. 10.1016/0741-8329(94)00072-7 7772264

[10] Domingo Z Bradley JK Blamire AM Brindle K Styles P Rajagopalan B. Diffusion weighted imaging and magnetic resonance spectroscopy in a low flow ischaemia model due to endothelin induced vasospasm. NMR Biomed. (2000) 13:154–62. 10.1002/1099-1492(200005)13:3<154::aid-nbm620>3.0.co;2-w 10861995

[11] Vink R McIntosh TK Demediuk P Weiner MW Faden AI. Decline in intracellular free Mg2+ is associated with irreversible tissue injury after brain trauma. J Biol Chem. (1988) 263:757–61. 10.1016/s0021-9258(19)35418-3 3335524

[12] Handa Y Kubota T Tsuchida A Kaneko M Caner H Kobayashi H. Effect of systemic hypotension on cerebral energy metabolism during chronic cerebral vasospasm in primates. J Neurosurg. (1993) 78:112–9. 10.3171/jns.1993.78.1.0112 8416225

[13] Vanhamme L van den Boogaart A Huffel SV. Improved method for accurate and efficient quantification of MRS data with use of prior knowledge. J Magn Reson. (1997) 129:35–43. 10.1006/jmre.1997.1244 9405214

[14] Liu Y Gu Y Yu X. Assessing tissue metabolism by phosphorous-31 magnetic resonance spectroscopy and imaging: a methodology review. Quant Imaging Med Surg. (2017) 7:707–26. 10.21037/qims.2017.11.03 29312876 PMC5756783

[15] Beer M Seyfarth T Sandstede J Landschütz W Lipke C Köstler H. Absolute concentrations of high-energy phosphate metabolites in normal, hypertrophied, and failing human myocardium measured noninvasively with 31P-SLOOP magnetic resonance spectroscopy. J Am Coll Cardiol. (2002) 40:1267–74. 10.1016/s0735-1097(02)02160-5 12383574

[16] Albers MJ Krieger MD Gonzalez-Gomez I Gilles FH McComb JG Nelson MD. Proton-decoupled 31P MRS in untreated pediatric brain tumors. Magnet Reson Med. (2005) 53:22–9. 10.1002/mrm.20312 15690498

[17] Wallimann T Wyss M Brdiczka D Nicolay K Eppenberger HM. Intracellular compartmentation, structure and function of creatine kinase isoenzymes in tissues with high and fluctuating energy demands: the ‘phosphocreatine circuit’ for cellular energy homeostasis. Biochem J. (1992) 281:21–40. 10.1042/bj2810021 1731757 PMC1130636

[18] Garnett MR Corkill RG Blamire AM Rajagopalan B Manners DN Young JD. Altered cellular metabolism following traumatic brain injury: a magnetic resonance spectroscopy study. J Neurotraum. (2001) 18:231–40. 10.1089/08977150151070838 11284544

[19] Pinggera D Steiger R Bauer M Kerschbaumer J Luger M Beer R. Cerebral energy status and altered metabolism in early severe TBI: first results of a prospective 31P-MRS feasibility study. Neurocrit Care. (2020) 34:432–40. 10.1007/s12028-020-01042-x 32617851

[20] Pinggera D Steiger R Bauer M Kerschbaumer J Beer R Rietzler A. Repeated 31P-magnetic resonance spectroscopy in severe traumatic brain injury: insights into cerebral energy status and altered metabolism. J Neurotraum. (2021) 38:2822–30. 10.1089/neu.2021.0143 34235953

[21] Stovell MG Mada MO Helmy A Carpenter TA Thelin EP Yan J-L. The effect of succinate on brain NADH/NAD+ redox state and high energy phosphate metabolism in acute traumatic brain injury. Sci Rep. (2018) 8:11140. 10.1038/s41598-018-29255-3 30042490 PMC6057963

[22] Yoshino A Hovda DA Kawamata T Katayama Y Becker DP. Dynamic changes in local cerebral glucose utilization following cerebral concussion in rats: evidence of a hyper- and subsequent hypometabolic state. Brain Res. (1991) 561:106–19. 10.1016/0006-8993(91)90755-k 1797338

[23] Pinggera D Luger M Bürgler I Bauer M Thomé C Petr O. Safety of early MRI examinations in severe TBI: a test battery for proper patient selection. Front Neurol. (2020) 11:219. 10.3389/fneur.2020.00219 32373042 PMC7179696

[24] Schubert GA Seiz M Hegewald AA Manville J Thomé C. Acute hypoperfusion immediately after subarachnoid hemorrhage: a xenon contrast-enhanced CT study. J Neurotraum. (2009) 26:2225–31. 10.1089/neu.2009.0924 19929373

[25] Steiger R Walchhofer L-M Rietzler A Mair KJ Knoflach M Glodny B. Cerebral phosphorus magnetic resonance spectroscopy in a patient with giant cell arteritis and endovascular therapy. Case Rep Radiol. (2018) 2018:7806395. 10.1155/2018/7806395 30510831 PMC6230418

[26] Weber-Fahr W Englisch S Esser A Tunc-Skarka N Meyer-Lindenberg A Ende G. Altered phospholipid metabolism in schizophrenia: a phosphorus 31 nuclear magnetic resonance spectroscopy study. Psychiatry Res Neuroimaging. (2013) 214:365–73. 10.1016/j.pscychresns.2013.06.011 24045051

[27] Shi X Carlson PJ Sung Y Fiedler KK Forrest LN Hellem TL. Decreased brain PME/PDE ratio in bipolar disorder: a preliminary 31P magnetic resonance spectroscopy study. Bipolar Disord. (2015) 17:743–52. 10.1111/bdi.12339 26477793 PMC5495548

[28] Kato T Inubushi T Kato N. Magnetic resonance spectroscopy in affective disorders. J Neuropsychiatry Clin Neurosci. (1998) 10:133–47. 10.1176/jnp.10.2.133 9608402

[29] VINK R FADEN AI McINTOSH TK. Changes in cellular bioenergetic state following graded traumatic brain injury in rats: determination by phosphorus 31 magnetic resonance spectroscopy. J Neurotraum. (1988) 5:315–30. 10.1089/neu.1988.5.315 3249310

[30] Vink R Golding EM Williams JP. McIntosh^*^ TK. Blood glucose concentration does not affect outcome in brain trauma. J Cereb Blood Flow Metabolism. (1997) 17:50–3. 10.1097/00004647-199701000-00007 8978386

[31] Ishige N Pitts LH Berry I Nishimura MC James TL. The effects of hypovolemic hypotension on high-energy phosphate metabolism of traumatized brain in rats. J Neurosurg. (1988) 68:129–36. 10.3171/jns.1988.68.1.0129 3335898

[32] Maudsley AA Govind V Levin B Saigal G Harris L Sheriff S. Distributions of magnetic resonance diffusion and spectroscopy measures with traumatic brain injury. J Neurotraum. (2015) 32:1056–63. 10.1089/neu.2014.3505 25333480 PMC4504344

[33] Stovell MG Mada MO Carpenter TA Yan J-L Guilfoyle MR Jalloh I. Phosphorus spectroscopy in acute TBI demonstrates metabolic changes that relate to outcome in the presence of normal structural MRI. J Cereb Blood Flow Metab. (2020) 40:67–84. 10.1177/0271678x18799176 30226401 PMC6927074

[34] Stovell MG Yan J-L Sleigh A Mada MO Carpenter TA Hutchinson PJA. Assessing metabolism and injury in acute human traumatic brain injury with magnetic resonance spectroscopy: current and future applications. Front Neurol. (2017) 8:426. 10.3389/fneur.2017.00426 28955291 PMC5600917

